# Removal of Toxic Copper Ion from Aqueous Media by Adsorption on Fly Ash-Derived Zeolites: Kinetic and Equilibrium Studies

**DOI:** 10.3390/polym13203468

**Published:** 2021-10-09

**Authors:** Gabriela Buema, Luisa-Maria Trifas, Maria Harja

**Affiliations:** 1 National Institute of Research and Development for Technical Physics, 47 Mangeron Boulevard, 700050 Iasi, Romania; gbuema@phys-iasi.ro; 2 Faculty of Chemical Engineering and Environmental Protection, “Gheorghe Asachi” Technical University of Iasi, 73 Prof.dr.doc. Dimitrie Mangeron Street, 700050 Iasi, Romania; trifas.luisa@yahoo.com

**Keywords:** copper adsorption, fly ash, NaOH treatment, zeolite

## Abstract

This study investigated the adsorption capacity of one material based on the treatment of fly ash with sodium hydroxide as a novel adsorbent for toxic Cu^2+^ ion removal from aqueous media. The adsorbent was obtained through direct activation of fly ash with 2M NaOH at 90 °C and 6 h of contact time. The adsorbent was characterized by recognized techniques for solid samples. The influence of adsorption parameters such as adsorbent dose, copper initial concentration and contact time was analyzed in order to establish the best adsorption conditions. The results revealed that the Langmuir model fitted with the copper adsorption data. The maximum copper adsorption capacity was 53.5 mg/g. The adsorption process followed the pseudo-second-order kinetic model. The results indicated that the mechanism of adsorption was chemisorption. The results also showed the copper ion removal efficiencies of the synthesized adsorbents. The proposed procedure is an innovative and economical method, which can be used for toxicity reduction by capitalizing on abundant solid waste and treatment wastewater.

## 1. Introduction

Copper ion is considered a toxic substance [1], whose health effects include stomachache, lung cancer and neurotoxicity. Copper and its compounds are widely used in many industries, such as: metal finishing, electroplating, plastics and etching [2]. A variety of traditional techniques for water treatment have been shown to be capable of removing copper from wastewater, including chemical precipitation, membrane filtration and ion exchange [3]. Adsorption can be explained as a separation process in which the components of a fluid phase are transferred to the surface of a solid material [4,5]. The adsorption process is the most used technique due to its efficiency and simplicity [6,7,8]. Several adsorbents have been tested in order to remove copper ion, such as activated carbon [8,9,10], clays [11], apatite [12], composite carbon-silica [13], magnetic materials [14,15,16], silica gel [17], hydroxyapatite [18], zeolites [19] and fly ash [5,20]. Over time, fly ash has been investigated by many researchers as an adsorbent for heavy metal removal [21,22]. The fly ash produced by coal combustion is a mixture of oxides with negative surface charge. The chemical composition of fly ash produced from different types of coal has been listed in the literature [22,23]. In accordance with ASTM C618, fly ash can be classified into two classes, depending on the sum of SiO_2_, Al_2_O_3_ and Fe_2_O_3_ [24]. Generally, the amount of alkalis (combined sodium and potassium) and sulphates are higher in Class C than in Class F. Ash is one of the most complex materials that can be characterized. There are approximately 316 individual minerals and 188 mineral groups that have been identified in different ashes [25]. For most ashes, the major phases consist of mullite, quartz and hematite [26].

The mineralogical classification system divides ash into four types—pozzolanic (*P*), inert (I), active (A) and mixed (M)—based on the distinct behavior between (1) the vitreous phase, (2) quartz + mullite and (3) the sum of other mineral phases, such as Fe–Ca–Mg–K–Na–Ti–Mn oxyhydroxides, sulphates, carbonates and silicates.

Fly ash, as an adsorbent, is associated with some problems, such as a small surface area (which leads to low adsorption capacities) and low storage [20]. On the other hand, the production of fly ash is an environmental problem; there are on-going efforts to find a use for this waste [27].

Dinah et al. have shown that the ash can be treated with alkaline solutions [28]. Obtaining these types of materials represents one of the most important applications of fly ash [29].

One intensive application of fly ash is as an adsorbent for heavy metal removal from aqueous media [22]. In the actual scientific context, it is underlined that implementing a cheap and highly efficient adsorbent to treat water contaminated with copper ions is an interesting challenge. In their investigations, many researchers tried to make fly ash more usable by its surface modification, subsequently trying to make useful products from the industrial waste. Therefore, fly ash treated with NaOH has become a subject of interest in heavy metal removal [20,30,31,32,33,34].

The authors have studied heavy metal removal onto fly ash and modified ash, and through over 10 years of experience have demonstrated that at low concentrations of activators, the performances of the obtained materials are limited [35]. This study demonstrates that an excellent adsorption capacity can be obtained, and for cheaper conditions if the other factors are appropriate. This research provides insights into the interaction mechanisms of modified fly ash with copper ions, facilitating the application of this type of material as an adsorbent. Unmodified fly ash shows a low adsorption capacity for Cu^2+^ ions, of approximately 14 mg/g [14]. Therefore, the main idea of the present study is to investigate the removal of Cu^2+^ toxic pollutants using an adsorbent based on fly ash treated with NaOH. The adsorbent is characterized using SEM (scanning electron microscopy), EDX (energy dispersive X–ray), XRD (X–ray diffraction), FTIR (Fourier transform infrared spectroscopy), and BET (Brunauer–Emmett–Teller surface area) analyses. Copper ion adsorption is examined within the adsorbent dose, initial concentration and contact time from an aqueous media. The equilibrium adsorption data are analyzed using two adsorption isotherm models: Langmuir and Freundlich. Two kinetic models are applied: pseudo-first-kinetic and pseudo-second-order.

The data obtained in this study demonstrate that the low-cost and easy synthesis of this new material based on the treatment of raw fly ash with sodium hydroxide in combination with its properties represents a promising adsorbent for copper ion removal.

## 2. Materials and Methods

### 2.1. Materials

The starting material used for the synthesis of the adsorbent was fly ash collected from the thermoelectrical power plant Holoca (Iasi, Romania). All the reagents used in this study were purchased from Chemical Company (Iasi, Romania) and did not require further purification.

### 2.2. Preparation of Adsorbent

To fabricate the adsorbent, a solution of NaOH (2M) and fly ash was prepared in a mixture of 3:1 ratio and stirred for 6 h at 90 °C. Afterwards, the resulting adsorbent was separated from the solution and rinsed several times with distilled water until it achieved a neutral pH. Then, it was dried at 60 °C for 24 h and stored.

### 2.3. Material Characterization

The following instrumental studies were performed in order to characterize the prepared adsorbent: SEM, EDX, XRD, FTIR and BET. The SEM and EDAX analyses were determined with a Quanta 3D instrument AL99/D8229 (FEI Company, Hillsboro, OR, USA). X–ray powder diffraction was conducted using an X’Pert PRO MRD X–ray diffractometer (PANalytical, Almelo, The Netherlands). The experiment was recorded by monitoring the diffraction pattern appearing in the 2θ range from 10° to 90°. Infrared absorption spectra (400–4000 cm^−1^) were analyzed with a Thermo Scientific Nicolet 6700 FT-IR spectrometer (Thermo Fisher Scientific, Waltham, MA, USA). BET surface area analysis was performed via Quantachrome instruments (Boynton Beach, FL, USA).

### 2.4. Batch Adsorption Experiments

Adsorption experiments were performed at room temperature, pH 5, at a shaking frequency of 300 rpm. Sample aliquots were collected after performing adsorption tests; the absorbance was measured at 390 nm wavelength using rubeanic acid [36]. Table 1 presents the adsorption experiments’ conditions. A Buck Scientific spectrophotometer (Buck Scientific, East Norwalk, CT, USA) was used for copper ion determination.

The adsorption capacity of the adsorbent, q (mg/g), was calculated based on Equation (1), and the adsorption efficiency was determined using Equation (2):

(1) $$q=\frac{\left(C_{i}-C_{e}\right)V}{m}$$

(2) $$\mathrm{Adsorption}\;\mathrm{efficiency},\;\%=\frac{\left(C_{i}-C_{e}\right)}{C_{i}}\times 100$$

where C_i_ (mg/L) represent the initial concentration of Cu^2+^ solution, C_e_ (mg/L) is the concentration of Cu^2+^ solution at equilibrium, V (L) is the volume of solution and m (g) is the quantity of the adsorbent used.

## 3. Results

### 3.1. Characterization of Adsorbent

**Figure 1:**
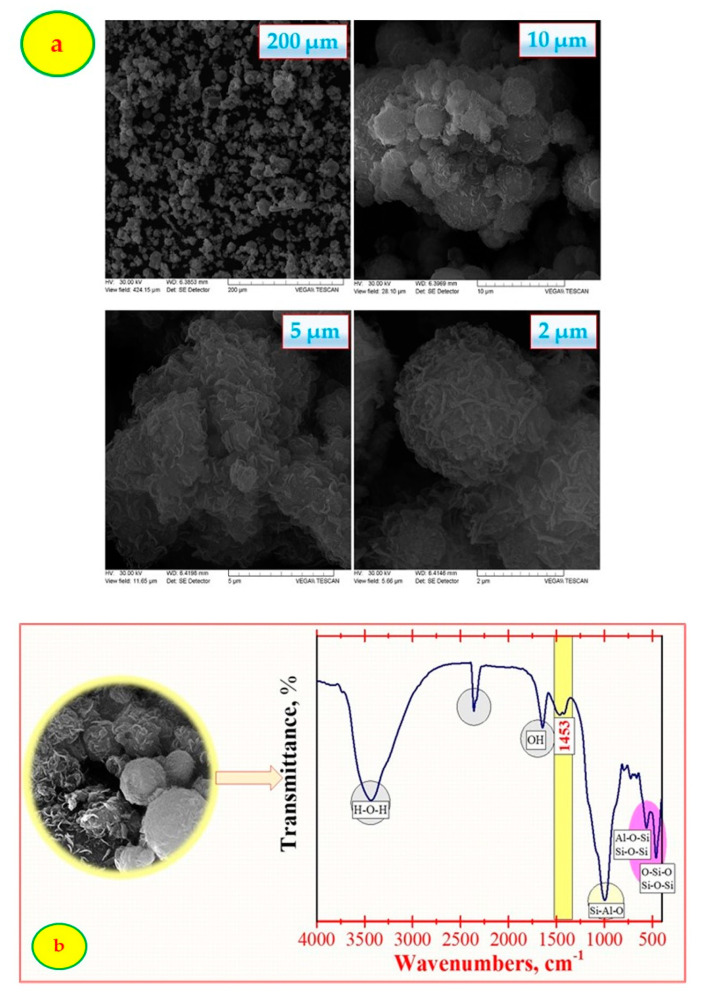

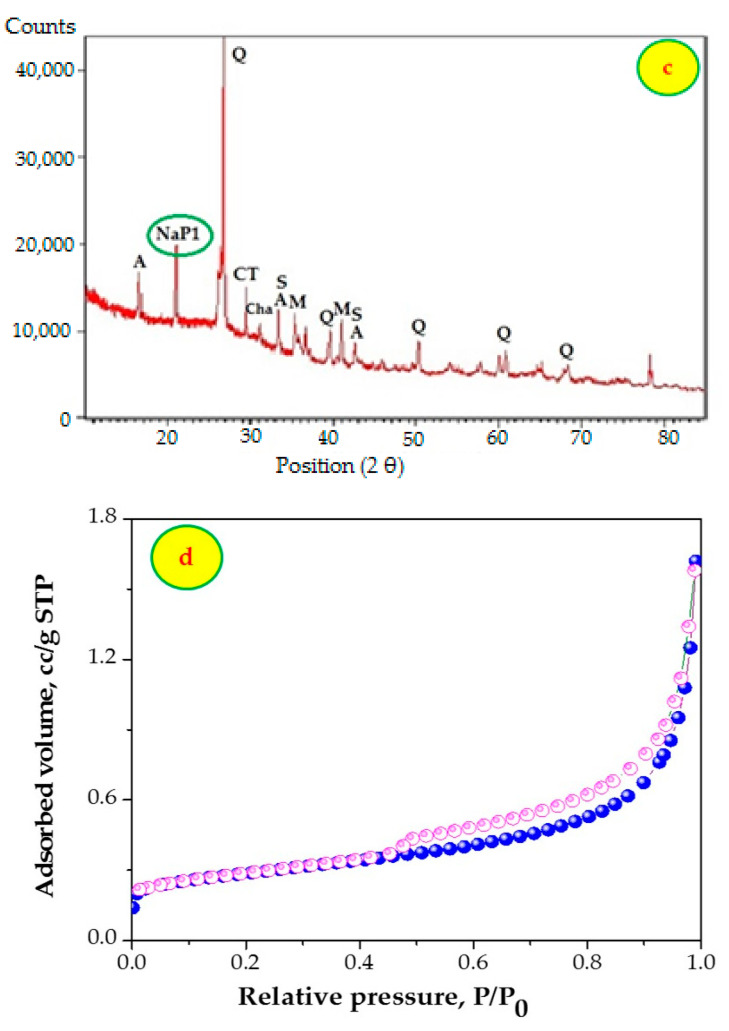
Characterization of the adsorbent: (**a**) SEM analysis; (**b**) FTIR analysis; (**c**) XRD analysis; (**d**) BET analysis.

SEM analysis was used in order to identify the surface morphology of the synthesized material, Figure 1a. A significant change in the material morphology was observed after treatment with NaOH at 90 °C, as confirmed by the SEM images. This change may be attributed to the chemical method by which NaOH was diffused into the structure of the fly ash.

The chemical composition of the material (Table 2), determined by EDX, indicated that the adsorbent contains Si, O, Al, Fe, Ca and Mg as well as small quantities of Ti, K and Na. The SiO_2_/Al_2_O_3_ ratio was approximately 1.3, which is in the range of NaP–type zeolites [37].

According to Figure 1b, the main functional groups were found at 3441 cm^−1^, 2362 cm^−1^, 1453 cm^−1^, 1645 cm^−1^, 1054 cm^−1^, 558 cm^−1^ and 466 cm^−1^. The peak at 2362 cm^−1^ can be assigned to the hydration water. The peak at 1453 cm^−1^ indicates that the sample included a GIS–NaP1 phase, a fact additionally demonstrated through the XRD analysis (Figure 1c). XRD analysis was interpreted according to the high-quality investigations performed by Treacy and Higgins [38].

The BET surface area of the synthesized adsorbent (Figure 1d) was found to be ~38 m^2^/g, while the total pore volume was 0.119 cm^3^/g. The results obtained indicate a higher surface area when fly ash is treated with NaOH that demonstrate a good adsorption capacity for Cu^2+^ ion removal.

The chemical composition of the material, determined by EDX measurement, is listed in Table 2. The detection limit of the device was 0.08% wt.

**Figure 2:**
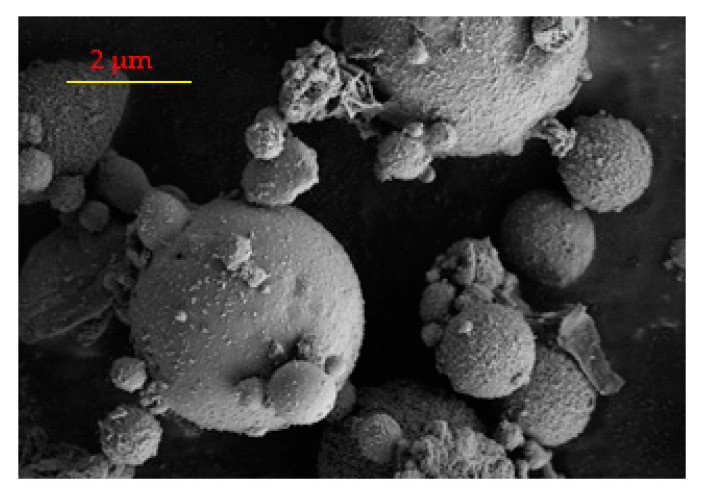
SEM image for raw fly ash.

For comparison, the results regarding the SEM and EDAX analysis for raw fly ash were included (Figure 2 and Table 3).

The fly ash from CET II Holboca Iasi contains: Cu = 298 ppm, Ni = 123 ppm, Cr = 127 ppm. Other toxic metals were also presented in our previous paper [39].

The SEM image of fly ash reveals that the sample has spherical particles and small quantities of irregular shapes of the particles. On the other hand, the EDX results show differences between the composition of fly ash and synthesized material. For example, the content of Na increased from 0.79% to 3.42%.

### 3.2. Adsorption Experiments

**Figure 3:**
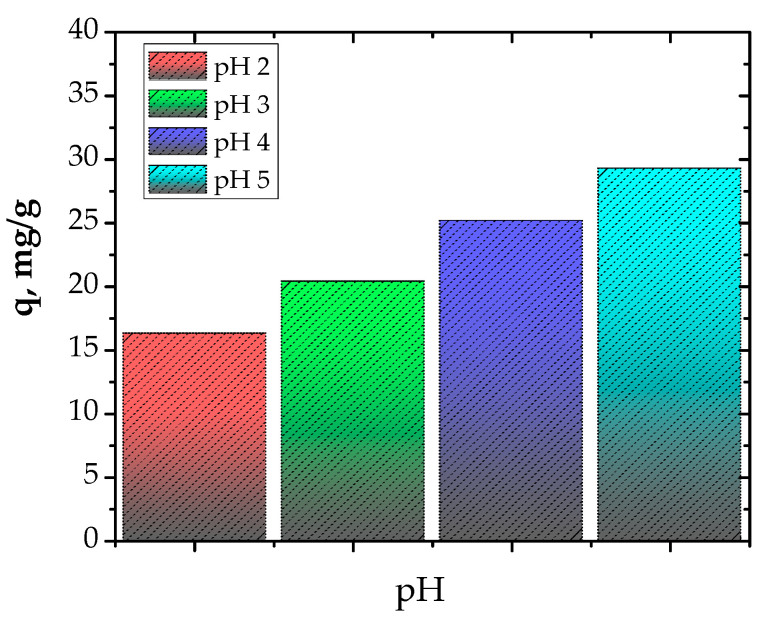
Influence of pH (contact time, 24 h; initial Cu^2+^ concentration, 300 mg/L; adsorbent dose, 1 g/100 mL; temperature, 25 °C).

The pH value is very important in the adsorption process. First of all, the influence of pH on the adsorption capacity of Cu^2+^ ion by the prepared material was investigated at pH values of 2, 3, 4 and 5 (Figure 3). The optimum pH value was found to be 5. Consequently, for further investigation regarding the influence of adsorbent dose, the initial Cu^2+^ concentration and the contact time, the pH value was kept constant at 5 in order to avoid Cu^2+^ precipitation.

**Figure 4:**
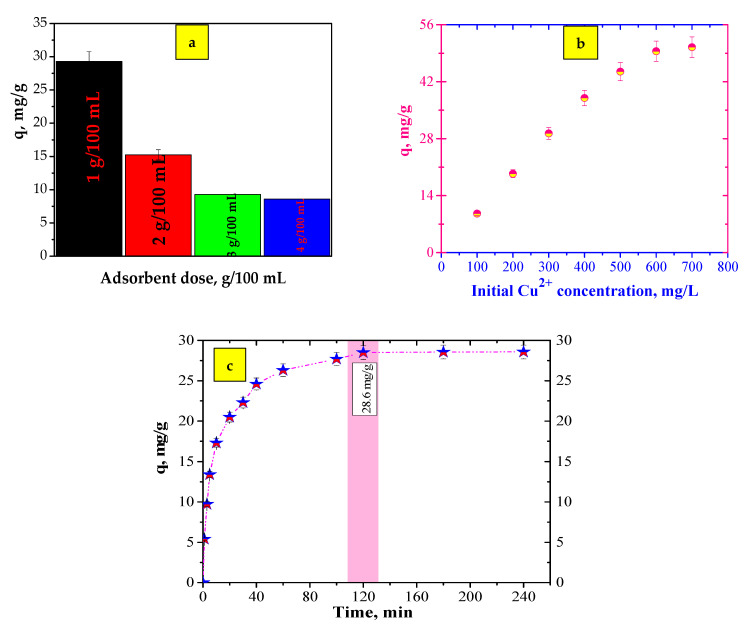
(**a**) Influence of adsorbent dose (pH, 5.0; contact time, 24 h; initial Cu^2+^ concentration, 300 mg/L; temperature, 25 °C); (**b**) Influence of initial concentration (pH, 5.0; contact time, 24 h; adsorbent dose, 1 g/100 mL; temperature, 25 °C); (**c**) Influence of contact time (pH 5.0; adsorbent dose, 1 g/100 mL; contact time, 240 min; initial Cu^2+^ concentration, 300 mg/L; temperature, 25 °C).

Adsorption experiments were conducted by varying the adsorbent dose, the initial Cu^2+^ concentration and the contact time. The results regarding the influence of the adsorbent dose, the initial concentration and the contact time are presented in Figure 4.

The influence of the adsorbent dose has a great effect on the adsorption process. The adsorbent dose influences the number of active sites available for the adsorption of pollutants [40]. The influence of the adsorbent dose on the adsorption capacity of the prepared adsorbent was investigated (Figure 4a). The effect of the adsorbent dose was performed using four dosages of the adsorbent (1–4 g/100 mL). According to Figure 4a, the adsorption capacity decreased as the adsorbent dose was increased from 1 g/100 mL to 4 g/100 mL. This fact may be attributed to the diminution of the adsorbent surface available to Cu^2+^ ions due to the agglomeration or aggregation of binding sites. The results indicated that 1 g/100 mL of synthesized material can be used for further experiments.

The initial Cu^2+^ ion concentration was studied at 100, 200, 300, 400, 500, 600 and 700 mg/L. Figure 4b presents the adsorption capacity of Cu^2+^ against the initial Cu^2+^ concentration. It is obvious that the adsorption capacity is affected by initial concentration values; thus, the adsorption capacity increased proportionally with the investigated parameter. The adsorption capacity rapidly increased when the initial concentration changed from 100 to 500 mg/L and reached the maximum adsorption capacity in the range of 600–700 mg/L. This aspect can be attributed to an increase in the driving force of Cu^2+^ ion toward the surface of synthesized material [41].

In order to study the influence of contact time on the Cu^2+^ adsorption capacity of the prepared adsorbent, the contact time was changed from 1 to 240 min. The results of the contact time are shown in Figure 4c. In the initial stage (≤ 40 min), the adsorption capacity rapidly increased due to the number of free available adsorption sites. As the contact time increased, the adsorption capacity tended to reach equilibrium. The adsorbent reached the maximum adsorption capacity of 28.6 mg/g within 120 min.

#### 3.2.1. Adsorption Isotherms

**Figure 5:**
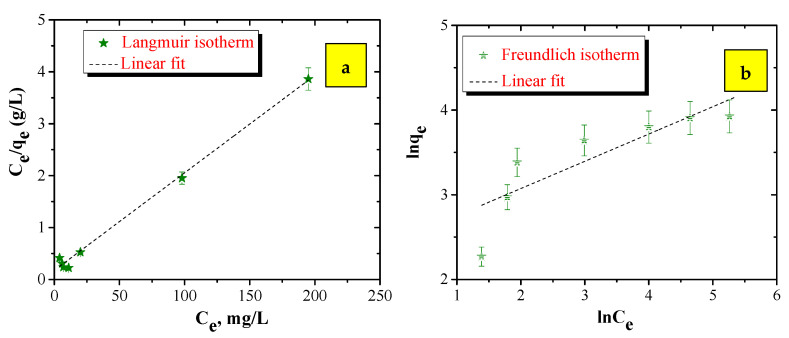
Langmuir (**a**) and Freundlich (**b**) isotherm plots for the adsorption of Cu^2+.^

The evaluation of adsorption isotherms was applied in order to ascertain the relationships between the prepared adsorbent and Cu^2+^ ion. The adsorption isotherm was analyzed using the Langmuir and Freundlich models [42]. All parameters corresponding to these models were calculated from the linear dependencies C_e_ vs. C_e_/q_e_ and lnC_e_ vs. lnq_e_. The plots for Cu^2+^ adsorption are displayed in Figure 5.

The values of the parameters corresponding to each isotherm model are summarized in Table 4.

The results displayed in Table 4 suggest that the adsorption of Cu^2+^ can be explained better by the Langmuir model (R^2^ ≥ 0.99), with a maximum amount of copper adsorbed of 53.5 mg/g. According to the Langmuir model, the theoretical maximum adsorption capacity and the maximum adsorption capacity of the adsorbent from the adsorption experiment were similar. The correlation coefficient of the other isotherm plot was relatively low, R^2^ = 0.7413.

**Figure 6:**
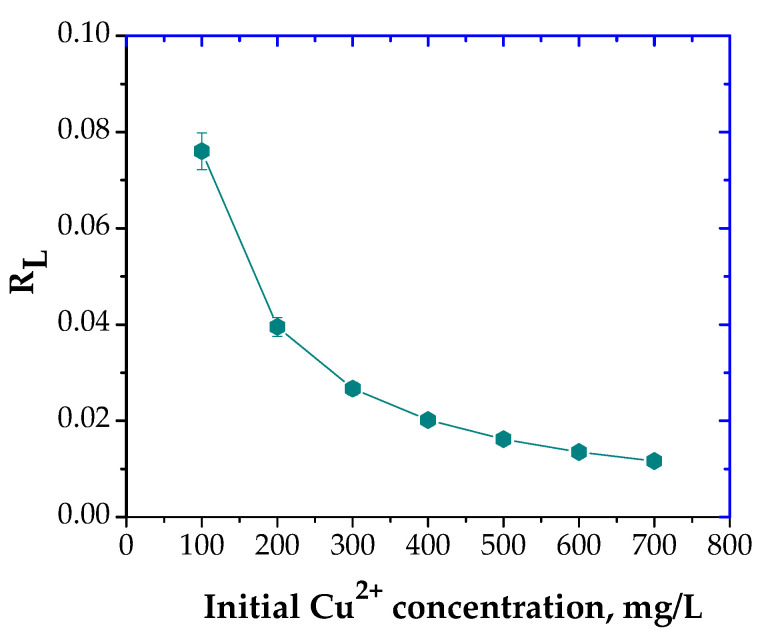
Plot of separation factor vs. initial Cu^2+^ initial concentration.

In addition, the fundamental characteristic and practicability of the Langmuir isotherm regarding a dimensionless constant separation factor or equilibrium parameter, R_L_, suggest that the adsorption process of Cu^2+^ using the prepared material was favorable, Figure 6, in accord with literature [43].

#### 3.2.2. Kinetics of the Adsorption Process

Among the kinetic models developed, the pseudo-first-order and pseudo-second-order kinetic models were used to study the adsorption process of Cu^2+^ ions. The equations for the pseudo-first-order kinetic model and the pseudo-second-order kinetic model are listed in Equations (3) and (4), respectively.

(3) $$\log\left(q_{e}-q_{t}\right)=\log q_{e}-\frac{\left(k_{1}t\right)}{2.303}$$

(4) $$\frac{t}{q_{t}}=\frac{1}{k_{2}\times q_{e}{}^{2}}+\frac{t}{q_{e}}$$

where q_t_ is the amount of cadmium adsorbed per unit of adsorbent (mg/g) at time t, k_1_ is the pseudo-first-order rate constant (1/min) and k_2_ is the pseudo-second-order rate constant (g/mg min).

The slopes and intercepts of the linear plots were used in order to calculate the kinetic parameters for the adsorption of Cu^2+^ ion by prepared material. The obtained results are summarized in Table 5.

According to Table 5, the R^2^ values obtained after fitting the data suggest that the pseudo-second-order model is more suitable than the pseudo-first-order model for describing the Cu^2+^ adsorption. The strong fit with the pseudo-second-order kinetic model suggests a chemical adsorption

**Figure 7:**
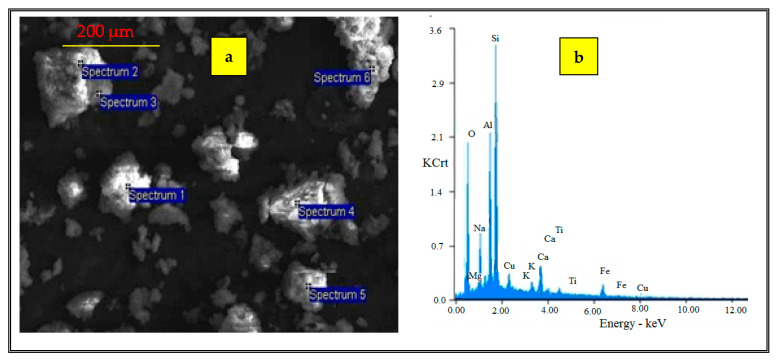
EDX analysis (**a**) after Cu^2+^ adsorption (**b**).

A supplementary EDX analysis after Cu^2+^ ion adsorption was performed (Figure 7). The data demonstrate that the toxic ions were attached to the surface of the prepared adsorbent.

Table 6 provides a comparison of the maximum adsorption capacity (q_max_) of treated fly ash with other previously reported studies from specialized literature.

According to Table 6, the material prepared in this study showed good adsorption capacity.

**Figure 8:**
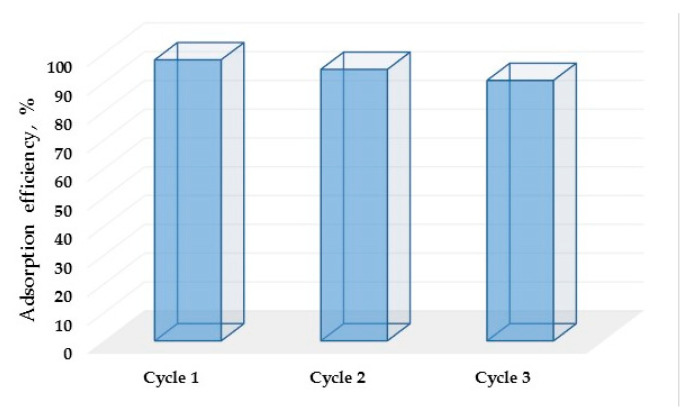
Adsorption efficiency of the synthesized material.

At the end of the study, the reusability investigation of the synthesized adsorbent was performed. Experimental cycles of adsorption/desorption were performed and fed with Cu^2+^ solution during the adsorption step and 0.1 [M] HNO_3_ during the desorption step. The obtained results are summarized in Figure 8.

From Figure 8, it is clear that the adsorption efficiency of the material drops a little after each cycle. This might be due to the amount of the adsorbent lost during the adsorption process. After three cycles the adsorption efficiency is still 90%, which made the synthesized adsorbent appropriate for use in wastewater treatment loaded with the copper ions.

## 4. Conclusions

The present research focused on the synthesis, characterization and applicability of one material based on the direct activation of fly ash with NaOH as an adsorbent for Cu^2+^ ion removal from aqueous media. The fly ash was synthesized under the following operating conditions: an NaOH concentration of 2M, NaOH; a fly ash ratio of 3:1; a temperature of synthesis of 90 °C; and a contact time of 6 h. In order to characterize the prepared adsorbent, SEM, EDX, XRD, FTIR and BET analyses were conducted.

The adsorption results were investigated based on two adsorption isotherms, specifically two kinetic models. The adsorption isotherms and kinetics were well described by the pseudo-second-order model and the Langmuir isotherm model. The maximum adsorption capacity for Cu^2+^ was 53.5 mg/g. The adsorption mechanism involved in the adsorption process was chemisorption.

This study demonstrated that fly ash may find application in the synthesis of low-cost adsorbents for toxic ion removal from aqueous media.

## Figures and Tables

**Table 1:** Adsorption experiment conditions.

Effect of Working Parameters	Adsorbent Dose	Initial Concentration	Contact Time
Effect of adsorbent dose	1 g/100 mL, 2 g/100 mL, 3 g/100 mL, 4 g/100 mL	300 mg/L	24 h
Effect of initial concentration	1 g/100 mL	100–700 mg/L	24 h
Effect of contact time	1 g/100 mL	300 mg/L	1–240 min

**Table 2:** EDX analysis, % atomic.

O	Na	Mg	Al	Si	K	Ca	Ti	Fe
31.98	3.42	1.02	13.29	16.97	0.68	2.31	1.03	8.02

**Table 3:** The chemical composition for raw fly ash, % atomic.

O	Na	Mg	Al	Si	K	Ca	Ti	Fe
43.32	0.79	0.62	19.19	30.81	1.75	1.15	1.54	3.05

**Table 4:** Adsorption isotherm constants.

Type of Isotherm	Equation	Linearization of Equation	Constants	R^2^
Langmuir	$q_{e}=\frac{q_{\max}\times K_{L}C_{e}}{1+K_{L}C_{e}}$	$\frac{C_{e}}{q_{e}}=\frac{1}{K_{L\;}\times q_{\max}}+\frac{C_{e}}{q_{\max}}$	q_max_ = 53.5 mg/g $K_{L}\;$= 0.094 L/g	0.9969
Freundlich	$q_{e}=K_{F}\times C_{e}{}^{1/n}$	$\log q_{e}=\left(\frac{1}{n}\right)\log C_{e}+\log K_{F}$	$K_{F}$ = 10.23 (mg/g)/(L/mg) 1/*n* = 0.3438	0.7413

Where: C_e_ (mg/L) is the concentration of metal in solution at equilibrium, q_e_ is the equilibrium metal adsorption capacity (mg/g), q_max_ is the maximum adsorption capacity (mg/g), K_L_ is the Langmuir constant (L/g), K_F_ is the Freundlich constant and 1/*n* is the heterogeneity factor.

**Table 5:** Comparison of the kinetic models for the adsorption of Cu^2+^.

q_e_, exp (mg/g)	Pseudo-First-Order Model		Pseudo-Second-Order Model		
	k_1_, (1/min)	R^2^	q_cal_, (mg/g)	k_2_ (g/mg min)	R^2^
28.6	0.0419	0.9732	30	0.0028	0.9986

**Table 6:** Comparison of adsorption capacity of various adsorbents for Cu^2+.^

Adsorbent.	q_max_, mg/g	Ref.
Nanosilica particles modified by Schiff base Ligands 3-methoxy salicylaldimine propyl triethoxysilane (MNS_1_)	3.73	[44]
Nanosilica particles modified by 5-bromo salicylaldimine propyl triethoxysilane	4.12	[44]
Nanosilica particles modified by 3-hydroxy salicylaldimine propyl triethoxysilane (MNS_3_)	5.82	[44]
Fe_3_O_4_@TETA	39.2	[45]
Fe_3_O_4_@DETA	44.2	[45]
Fe_3_O_4_@DAMP	52.3	[45]
Natural bentonite treated with H_2_SO_4_ (ARH)	17.24	[46]
Calcium homoionic clay (ARC)	18.18	[46]
Sodium homoionic clay (ARS)	24.39	[46]
Acid-treated bio-sorbent (ATB)	8	[47]
Untreated bio-sorbent (UTB)	19	[47]
Base treated bio-sorbent (BTB)	25	[47]
Detergent treated bio-sorbent (DTB)	28	[47]
Treated fly ash	53.5	This work

## Data Availability

The data presented in this study are available on request from the corresponding author.
